# TRIM32 promotes tumor immune evasion and impedes Anti–PD-1 treatment by inducing immunosuppressive macrophages in gastric cancer

**DOI:** 10.1186/s12967-025-06330-8

**Published:** 2025-10-29

**Authors:** Changming Wang, Xujun Zhu, Jun Wang, Zhiqing Hu, Pengcheng Xiang, Jian Xu, Jiapeng Xu, Qingping Cai

**Affiliations:** 1 Department of Gastrointestinal Surgery, Department of General Surgery, Shanghai East Hospital, Tongji University School of Medicine, 150 Jimo Road, Shanghai, 200120 China; 2 Department of Gastrointestinal Surgery, Shanghai Baoshan District Hospital of Integrated Traditional Chinese and Western Medicine, Shanghai University of Traditional Chinese Medicine, Shanghai, China

**Keywords:** TRIM32, Tumor-associated macrophages, Gastric cancer, PDE9A

## Abstract

**Background:**

The tumor microenvironment (TME) in gastric cancer (GC) exhibits immunosuppressive features that facilitate tumor advancement and obstruct the effectiveness of immunotherapy. The role of tripartite motif 32 (TRIM32) in the TME has not been extensively studied.

**Methods:**

GC mouse model was utilized along with flow cytometry analysis, transwell assays, and immunohistochemistry to investigate the impact of TRIM32 on tumor progression and macrophage. To uncover the mechanisms by which TRIM32 operates within the GC microenvironment, various molecular and biochemical methods were utilized, including RNA-sequencing, western blotting, quantitative reverse transcription-polymerase chain reaction, coimmunoprecipitation, and immunofluorescence.

**Results:**

TRIM32 originating from tumors was found to be linked to poor prognosis and notably associated with tumor-associated macrophages (TAMs) in. In vitro experiments revealed that TRIM32 induced TAMs recruitment and M2-like polarization. Mechanismly, TRIM32 interacted with Phosphodiesterase 9 A (PDE9A) and activated the downstream phosphatidylinositol-3-kinase/protein kinase B (PI3K/Akt) signaling pathway. Additionally, the reprogramming of TAMs by TRIM32 diminished the resistance to anti-PD-1 treatment in GC models.

**Conclusion:**

TRIM32/PDE9A axis promotes immune evasion in tumors and hinders the effectiveness of anti-PD-1 treatment by inducing TAMs recruitment and M2-like polarization in GC. This research provides insight into the role of TRIM32 in modulating tumor immunity and suggests that TRIM32 could be a promising target to overcoming resistance to anti-PD-1 therapy in GC.

**Supplementary Information:**

The online version contains supplementary material available at 10.1186/s12967-025-06330-8.

## Background

Gastric cancer (GC) is a deeply aggressive malignancy, with a notable number of new cases and deaths reported globally every year [1]. Early detection is crucial for successful treatment outcomes, as late-stage GC often leads to poor prognosis due to extensive invasion and metastasis. In addition to targeted therapy, tumor immunotherapy has surfaced as a promising alternative treatment, wherein the tumor microenvironment (TME) playing a crucial role in shaping its efficacy. Innovative strategies that address various immunomodulatory mechanisms within the TME possess the potential to notably enhance the prognosis for patients with GC [2].

The TME, consisting of various components such as cell types, soluble substances, and the extracellular matrix, is essential for the advancement of cancer [3]. Within the TME, tumor-associated macrophages (TAMs) serve as crucial immune cells that can assume either pro-inflammatory (M1) or anti-inflammatory (M2) states, depending on the signals they receive from the tumor environment [4]. The immunosuppressive nature of M2 macrophages, which promote immune tolerance and hinder anti-tumor immune responses within the GC TME, contributes significantly to tumor development [5]. Studies have identified specific mechanisms by which TAMs are recruited and polarized in GC [6, 7], such as the involvement of exosome circATP8A1 [8] and ETS-like transcription factor 4 [9] in promoting M2 polarization and tumor progression. Gaining insight into the molecular mechanisms associated with TAM polarization in GC may could lead to the development of targeted therapeutic approaches. Targeting gene expression changes that influence these polarization-related pathways could represent a promising direction for new therapeutic approaches in GC. By addressing the mechanisms that drive M2 macrophage polarization within the TME, researchers may uncover new opportunities for enhancing immunotherapy and improving outcomes for GC patients.

TRIM32, a member of the TRIM family, potentially influencing cell differentiation and carcinogenesis. TRIM32 has been implicated in carcinogenesis, with elevated levels of TRIM32 has been associated with the development of cancer, as increased levels of TRIM32 mRNA have been found in hepatocellular carcinoma, GC, and breast cancer, where it serves as a biomarker for prognosis [10–12]. Moreover, the overexpression of TRIM32 promoted cell proliferation rates, and induced drug resistance in cancer cells [10, 12]. Our earlier study found that TRIM32 was expressed at elevated levels in GC tissues and cell lines, which resulted in enhanced cell proliferation, migration, and invasion, as well as a poorer prognosis [13]. The presence of particular immune cell types within tumors is significantly linked to tumor proliferation, metastasis, drug resistance, and the overall prognosis for patients [14]. Research indicates that TRIM32 plays a role in the immune evasion of the HIV-1 virus [15]. It also regulates the innate immune response and the production of inducible nitric oxide synthase (iNOS) in macrophages to fight sepsis [16]. Moreover, the deficiency of TRIM32 homolog, TRIM59 has been reported to enhance M1 macrophage activation through the STAT1 signaling pathway in colorectal cancer [17]. The other TRIM32 homolog, TRIM56 also have been reported that overexpression promoted the polarization of M2 macrophages in glioma [18, 19]. Therefore, we hypothesized that the malignant progression of tumors and their resistance to drugs could be influenced by TRIM32 through its regulation of macrophage polarization.

In this study, we will integrate bioinformatics methodologies with both in vivo and in vitro experiments to investigate the how TRIM32 influences the polarization of macrophages and anti-PD-1 resistance, potentially providing insights into how TRIM32 drives tumor progression and revealing new therapeutic opportunities for GC.

## Methods

### Patient populations

A total of 118 patients with GC who underwent primary tumor debulking surgery at Shanghai East Hospital, Tongji University School of Medicine in Shanghai, China, between 1 April 2022 and 31 January 2024 were included in this study. Before being included in the study, all patients submitted written informed consent. The gathering and handling of clinical specimens for this particular research study were performed with the endorsement of the Ethics Committee at Shanghai East Hospital (approval number: 2023 − 159).

### Cell lines and cell culture

The human GC cell lines AGS (which are moderately differentiated), HGC27 (characterized as undifferentiated), THP-1 (human monocytic cells), along with the mouse GC cell line mouse forestomach carcinoma (MFC) and mouse macrophage cell line RAW264.7 were sourced from Cell Resource Center of the Chinese Academy of Sciences (Shanghai, China). These cell lines were cultured in RPMI-1640 medium (C11875500BT, Gibco, MA, USA) supplemented with 10% fetal bovine serum (FBS, BC-SE-FBS07, Bio-channel, Nanjing, China) at 37 °C supplemented with 5% CO_2_. A short tandem repeat (STR) analysis was utilized for authentication of the cell lines.

THP-1 cells were received treatment with phorbol 12-myristate 13-acetate (100ng/ml, 79346, Sigma-Aldrich, MI, USA) for a duration of 24 h to facilitate their differentiation into M0 macrophages [20]. To generate conditioned medium (CM), AGS, HGC27, and MFC cells were incubated in serum-free medium for 24 h, after which cell debris was eliminated from the CM via centrifugation.

### In vitro treatment with the PI3K/Akt activator or inhibitor

GC cells were grown in 6-cm cell dishes and then exposed to either 1 µM IPI-549 (S8330, Selleckchem, Houston, TX, USA) or 10 nM Insulin-like Growth Factor 1 (IGF-1, 100 − 11, PeproTech, Rocky Hill, NJ, USA) for 48 h, during which the culture medium was replaced every 24 h.

### Lentiviral vector construction and transfection

The PDE9A-overexpressing (ovPDE9A) lentivirus, PDE9A short hairpin RNA (shPDE9A) lentivirus, TRIM32 short hairpin RNA (shTRIM32) lentivirus and negative control (NC) were constructed and synthesized by Hanheng Biotechnology (Shanghai, China). TRIM32 and PDE9A short hairpin RNA were cloned into the pLKO.1 vector, while their cDNAs were inserted into the pCDH-CMV-MCS-EF1-puro vector for stable overexpression. H293T cells were transfected with these vectors and viral packaging plasmids (pMD2.G and psPAX2) using Lipofectamine 6000 (C0526, Beyotime, Shanghai, China). After 48 h, the virus supernatant was harvested, filtered, and incubated with target cells for 6 h at a 1:10 dilution with 10 µg/mL polybrene (H9268, Sigma, St Louis, MO, USA). The efficiency of transfection was confirmed through quantitative reverse transcription–polymerase chain reaction (qRT-PCR) and western blot analysis (WB). The sequences of shRNA utilized in this research were as follows. shTRIM32-1 sequence: GGGACUUUGGAGAGAAGUUAA, shTRIM32-2 sequence: GCAAGAUGUGGAGCUCCUUAA. shTRIM32-3 sequence: GGUGGAAAGCUUUGGUGUU, and shNC sequence UUCUCCGAACGAGUCACGU.

### Mice and animal experiments

All BALB/C nude mice (6 ~ 8 weeks, sex matched) were procured from Jiesijie lab (Shanghai, China). All procedures involving the mice were conducted in strict accordance with the Principles for the Utilization and Care of Vertebrate Animals and the Guide for the Care and Use of Laboratory Animals. The Ethics Committee of Shanghai East Hospital granted approval for all animal experiments (approval number: 2023 − 159).

Mice were injected subcutaneously (s.c.) with 10^6^ MFC cells on day 0. The dimensions of the tumors were evaluated using calipers, employing this formula to determine tumor volume (mm^3^): tumor volume = L*W^2^/2, where L is the long axis size and W is the vertical size. Finally, the tumor was weighed and photographed. A portion of the tumor tissue was processed into single-cell suspensions for the analysis of TAMs through flow cytometry.

For TAM depletion, clodronate liposomes (CL, F70101, FormuMax, California, USA) were used to reducing macrophages populations within the TME. Following the inoculation of tumor cells, the mice received intraperitoneally injections of 1 mg CL twice a week for 2 weeks. After the tumor challenge, mice were administered intraperitoneally with 10 mg/kg of mouse PD-1 antibody (anti–PD-1, BE0273, BioXcell, NewHampshire, USA) or PBS 5 times over 14 days.

### Single cell suspension preparation

To create a suspension of single cells, tumor tissues were diced into approximately 1 mm³ fragments within RPMI-1640 medium supplemented with 2% FBS. They were then digested for 40 min utilizing the MACS Tumor Dissociation Kit (130-095-929, Miltenyi Biotec, Bergisch Gladbach, Germany) on a gentleMACS™ Dissociator. After digestion, the cells were passed through a 70 μm filter, centrifuged at 500 g for 10 min, re-suspended in MACS buffer (1x PBS containing 2mM EDTA and 0.5% BSA), and subsequently counted.

### Transwell cell migration assay

Migration capacity was assessed in THP-1 cells (2 × 10 ^5^) by placing them in the upper chamber of a transwell membrane, while CM from AGS/HGC27 cells was added to the lower chamber. After migration, the cells that reached the bottom chamber were fixed in 4% formaldehyde for 20 min and subsequently stained with 0.5% crystal violet for analysis. Using an inverted microscope, observation and photography of five random fields at 200× magnification was conducted.

### Flow cytometry

To detect macrophage cell marker expression, 3 × 10 ^5^ cells were incubated with mouse antibodies specific to cell markers for 30 min at 4 °C, after which they were washed two times with PBS. Viable cells were then filtered by utilizing forward and side scatter characteristics for gating. Fluorescence signals were detected and analyzed by a BD FACSC elesta Cell Analyzer (LSRII, BD Biosciences, San Jose, CA, USA).

### qRT-PCR assay

RNA was extracted from AGS and HGC27 cells utilizing RNAiso Plus Kits (9109, TaKaRa, Shiga, Japan) following the manufacturers’ direction. Reverse transcription of RNA into cDNA was carried out using the PrimeScriptTM RT Reagent Kit 036 A (TaKaRa), and and quantification was achieved using SYBR^®^ Premix Ex TaqTM II 820 A (TaKaRa). The expression levels were normalized to the geometric mean of the housekeeping gene GAPDH. Data analysis was conducted utilizing ABI7500 system software, and calculations were made employing using the 2^−ΔΔCt^ method. Below are the primer sequences utilized for PCR. TRIM32 5`-GTGGACTCGTCGGAGCC − 3` (forward) and TRIM32 5`-AGCTCAGAACTGAACAGCACA-3` (reverse). PDE9A 5`-TGCAACTCCAGCGACATCAT-3` (forward) and PDE9A 5`-TTGATGGCCACAGGTCTCAC–3` (reverse).

### WB analysis

WB analysis was conducted as described previously [13]. Cells were lysed using SDS buffer, and the protein concentration was quantified by Pierce BCA Protein Assay kit (23225, Thermo Fisher Scientific, Waltham, MA, USA). Quantified lysates were subjected to SDS-PAGE gels, followed by electrotransfer to PVDF membranes (IPVH00010, Millipore, MA, USA). Primary antibodies were incubated with the proteins at dilutions of 1:1,000 or 1:2,000 overnight. Following three washes with TBST for 10 min each, the samples were then incubated with secondary antibodies (diluted 1:2000) at room temperature for 2 h on a shaker. After an additional three washes using TBST, chemiluminescence development was monitored. Protein bands were analyzed using Image J (Version 1.8.0, NIH, USA). A complete list of antibodies can be found in Supplementary Table 1.

### Transcriptome sequencing

In this study, the total RNA was obtained from the transfected AGS cells (3 samples in the shNC group and 3 samples in the shTRIM32 group) using the TransZol™ Up Plus RNA Kit (ER501, Transgen, Beijing, China). To verify that the samples are appropriate for transcriptome sequencing, assessments of RNA sample purity, concentration, and integrity were performed utilizing the NanoDrop 2000 (Thermo Fisher Scientific) and the Agilent 2100 Bioanalyzer (Agilent, Santa Clara, CA). The sequencing libraries were prepared using the VAHTS^®^ Universal V8 RNA-seq Library Prep Kit for Illumina (Illumina, San Diego, CA, USA). The Qubit RNA Assay Kit (Q32855, Thermo Fisher Scientific) on a Qubit 4.0 was employed to measure the RNA concentration of the libraries. Following the manufacturer’s instructions, sequencing was carried out on an Illumina NovaSeq platform, employing the GRCh38.p13 genome reference sequences. Differential gene analysis between the shNC and shTRIM32 groups was conducted using DESeq2, focusing on significantly downregulated genes (log fold-change ≥ 1, P.adjust < 0.05) for KEGG pathway analysis (http://kegg.jp).

### Bioinformatics analysis

The association of TRIM32 copy number or expression with immune infiltration levels in GC macrophages, in addition to the expression of macrophage markers, was evaluated by employing both the TIMER database [21] (https://cistrome.shinyapps.io/timer/, http://timer.cistrome.org/) and The Cancer Genome Atlas (TCGA) database. For the correlation analysis, expression data were transformed using log2 (transcripts per million, TPM). To assess statistical correlations, Spearman’s rank correlation test was applied.

The effects of different expression levels of TRIM32 on overall survival were analyzed using Kaplan⁃Meier Plotter [22]. Kaplan-Meier survival analyses for disease outcomes utilizing TCGA dataset (*n* = 611) were performed through the online platform (www.kmplot.com). Patients were categorized into “low” and “high” expression groups based on median TRIM32 mRNA expression (in TPM). Statistical significance was calculated using the log-rank (Mantel-Cox) test, with a significance threshold established at *p < 0.05.

### Immunofluorescence assay

Cells were treated with 4% paraformaldehyde for 30 min at room temperature, followed by washing with 0.1% Triton-X-100 in phosphate-buffered saline (PBST) and incubation overnight at 4 °C in PBST containing 3% BSA. Tumor samples were prepared as 5 mm formalin-fixed, paraffin-embedded (FFPE) blocks, subjected to blocking with 3% H₂O₂ in methanol, and underwent antigen retrieval using citrate buffer. After washing, the sections were blocked with 5% BSA in PBS for 30 min at room temperature. Both tissue sections and cells were incubated with primary antibodies overnight at 4 °C, followed by washing and treatment with secondary antibodies for 1 h at 4 °C in the dark. DAPI was used to stain the nuclei, and immunofluorescence was examined with confocal microscopy (Leica SP5 Confocal Microscope, Leica). The antibodies employed are detailed in Supplementary Table 1.

### Co-immunoprecipitation (Co-IP) assay

When the AGS and HGC27 cells were reached 80–90% confluence, they were subjected to three washed with ice-cold PBS before being lysed on ice for 30 min using an immunoprecipitation lysis buffer. Clarification of the lysates was performed by centrifugation at 14,000×g for 10 min, after which the supernatant of the cell lysate was incubated overnight with the primary antibody. Subsequently, the cell lysates were mixed with Protein A/G-PLUS Agarose beads (sc-2003, Santa Cruz, Dallas, TX, USA) and incubated for 4 h. Following the processes of centrifugation and washing, the eluates were analyzed via SDS-PAGE and WB. All CO-IP experiments were conducted at 4 °C. All antibodies were listed in Supplementary Table 1.

### Statistical analysis

All statistical analyses were conducted using GraphPad Prism version 9.0 (GraphPad). Results are presented as means ± standard deviation (SD) from at least three separately experiments, each carried out in triplicate. Pearson correlation tested relationships between continuous variables. For comparing multiple groups, one-way ANOVA with Tukey’s post hoc test was applied, while a two-tailed Student’s t-test was utilized for comparisons between two groups. A p-value of less than 0.05 was considered to indicate statistical significance.

## Result

### High TRIM32 expression is linked to poor prognosis and TAM infiltration in GC patients

**Fig. 1:**
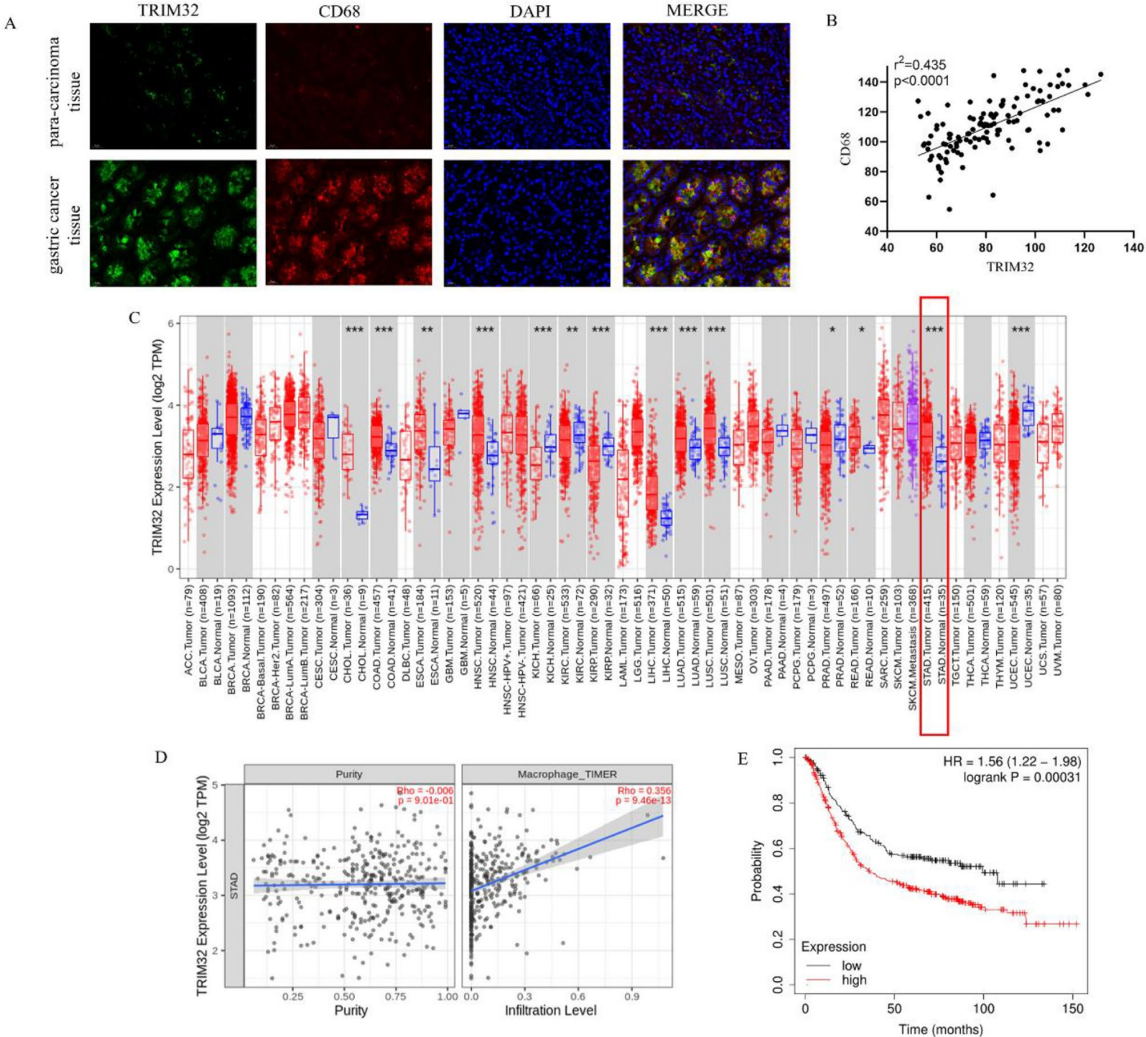
High levels of TRIM32 expression are linked to poor prognosis and increased macrophage abundance in GC patients. (**A-B**) Immunofluorescence analysis revealed that patients with elevated TRIM32 expression in GC tumor cells had significantly increased infiltration of CD68 + TAMs. (**A**) Representative images display TRIM32 expression and TAM infiltration (scale = 20 μm). (**B**) Data from 118 patients on average. TRIM32 and CD68 expressions were quantified using mean fluorescence intensities. The Pearson correlation between TRIM32 and CD68 expressions was significant (*n* = 118; *p* < 0.001, r^2^ = 0.435). (**C**) TRIM32 exhibited elevated expression levels in STAD tumor tissues. The pan-cancer expression pattern of TRIM32 in tumor tissue and corresponding nontumor tissues was acquired from the TIMER database; *p < 0.05, **p < 0.01, ***p < 0.001). (**D**) The relationships between TRIM32 expression and macrophage infiltration in GC were assessed using TIMER2.0. (E) The Kaplan-Meier survival curve suggested that TRIM32 has prognostic significance in STAD utilizing data from the TCGA cohort, which classified patients into high (*n* = 402) and low (*n* = 209) groups based on a median cutoff

We collected 118 GC samples from the Shanghai East Hospital Cohort. Supplementary Table 2 shows the clinicopathologic characteristics of our cohort. Immunofluorescence analysis revealed higher levels of TRIM32 protein in tumor tissues versus normal tissues (Fig. 1A). CD68, a marker for monocyte lineage, circulating macrophages, and tissue-resident macrophages, was found to be highly expressed [23]. A positive relationship was noted between elevated TRIM32 levels and the count of CD68 + cells (Fig. 1B). Subsequent analysis using TIMER database on TCGA data confirmed the elevated expression of TRIM32 in Stomach adenocarcinoma (STAD, Fig. 1C), and showed a positive association between TRIM32 expression and macrophage abundance (Fig. 1D), indicating a role of TRIM32 in promoting macrophage enrichment in GC. In the TCGA cohort, higher expression levels of TRIM32 were linked to poor clinical outcomes in patients with GC, as demonstrated by the overall survival analysis (Fig. 1E). These findings collectively suggest that high TRIM32 expression is associated with poor prognosis and more macrophages infiltration in GC patients.

### TRIM32 blockade inhibited recruitment and M2 polarization of macrophages

**Fig. 2:**
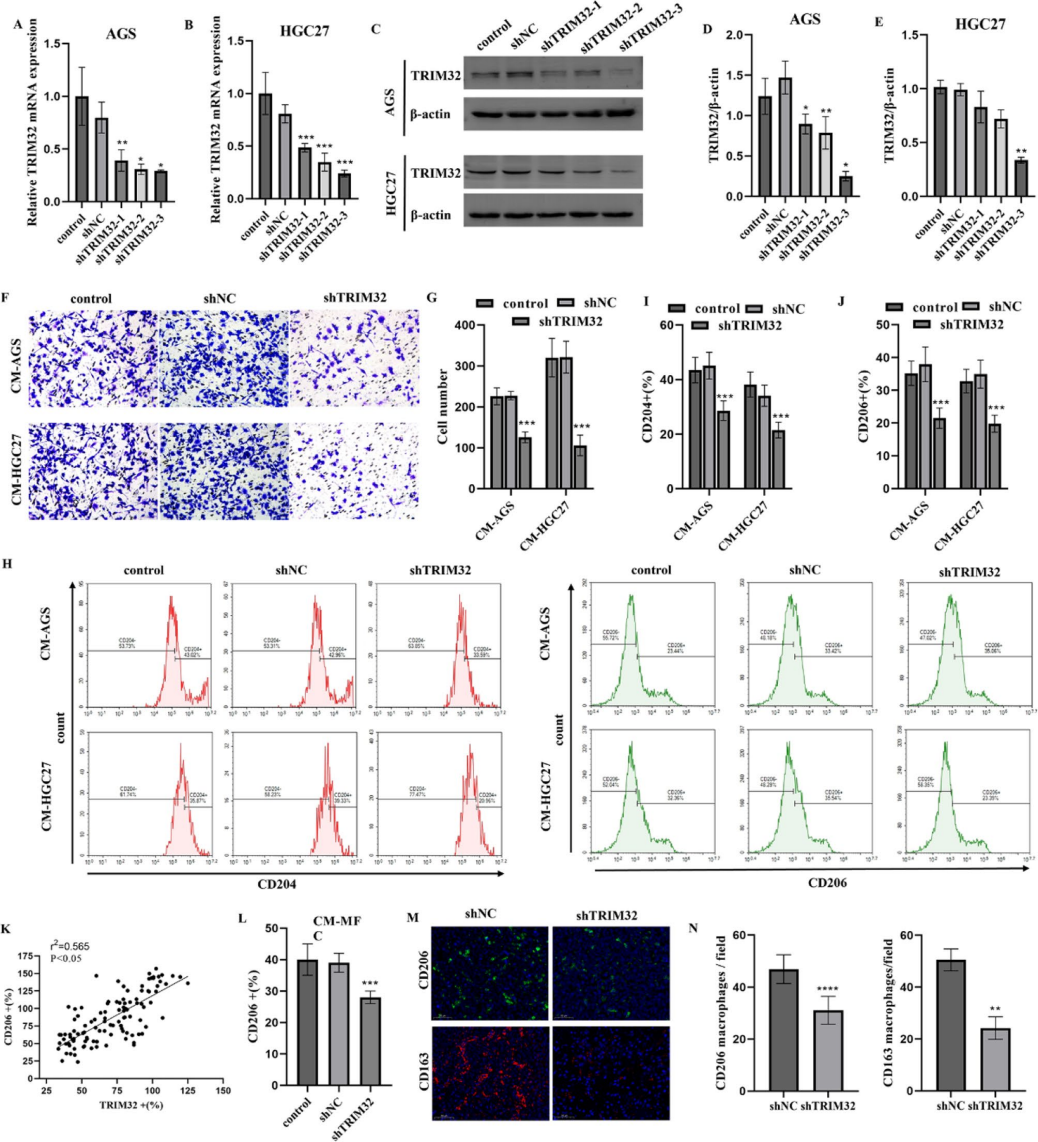
Blocking TRIM32 inhibited macrophage recruitment and M2 polarization in vitro. (**A-E**) Lentivirus carrying specific shRNA sequences significantly reduced TRIM32 expression in AGS and HGC27 cells, as verified by qRT-PCR (**A-B**) and Western blotting (**C-E**). (**F-G**) TRIM32 knockdown in these cells hindered tumor-induced TAM migration. After transfecting tumor cells with TRIM32 shRNA lentivirus for 48 h, CM was collected to culture macrophages. TAM migration was evaluated using a transwell assay, with (**F**) images of migrated cells and (**G**) average results from each group. ***p < 0.001 vs. shNC group. (**H-J**) PMA-stimulated THP-1 cells cultured in AGS/HGC27-derived CM showed reduced M2-like polarization with TRIM32 knockdown. Macrophage polarization was assessed by (**H**) flow cytometry and (**I-J**) average results from three experiments. (**K**) Patients with high TRIM32 expression in tumor cells of GC tissues showed markedly elevated infiltration of CD206 + TAMs by immunofluorescence analysis. Average results from 118 patients. The expressions of TRIM32 and CD206 were measured with mean fluorescence intensities, respectively. The pearson correlation between TRIM32 and CD208 expression (*n* = 118; *p* < 0.05, r^2^ = 0.565). (**L**) RAW264.7 cells cultured in MFC/shTRIM32-derived CM showed inhibited M2-like macrophage polarization. MFC cells were first transfected with TRIM32-knockdown lentivirus for 48 h, and CM was collected for TAM induction. The polarization ability of macrophage cells was assessed by Flow cytometry results and Average results from three independent experiments. (**M-N**) The knockdown of TRIM32 leads to a reduction in CD206 and CD163-positive cells within solid tumors. (**M**) Representative images of CD206 and CD163-positive cells (scale = 50 μm). (**N**) Average results from each group. Data with error bars are shown as mean ± SD. *P < 0.05, **P < 0.01, ***P < 0.001, ****P < 0.0001

To investigate the impact of TRIM32 on macrophages, stable TRIM32 knockdown was established in HGC27 and AGS GC cell lines (Fig. 2A-E). The transwell assay results demonstrated that migration of THP-1 cells was hindered by CM derived from shTRIM32 compared to shNC and control (Fig. 2F-G). Subsequently, THP-1 cells were treated with PMA to differentiate into M0 macrophages, then exposed to CM. Flow cytometry showed that TRIM32 knockdown decreased CD204 + and CD206 + M2-like macrophage polarization (Fig. 2H-J). The CM obtained from shTRIM32 cells also showed an increase in M1-like markers such as IL-1β, IL-12 A, and CD86, while simultaneously decreasing the levels of the M2-like marker Arg-1 (see Supplementary Figure S1A and B). Furthermore, immunofluorescence analysis of patient tissues indicated a positive relationship between the expression levels of TRIM32 and those of CD206 (Fig. 2K). Flow cytometry showed that the percentage of CD206 + RAW264.7 M2 macrophages were decreased in MFC/shTRIM32-derived CM (Fig. 2L). The mRNA expression of tumor-supportive macrophage genes, such as Arg-1, was reduced by shTRIM32-CM, whereas the expression of cytotoxic response genes IL-1β, IL-12 A, and CD86 was elevated by shTRIM32 cell supernatants (Supplementary Figure S1C). We assessed CD206 and CD163 expression to investigate TAM polarization in solid tumors and found significantly reduced M2-like TAMs in shTRIM32 groups (Fig. 2M-N). A cytolytic assay demonstrated that TRIM32 downregulation in AGS cells enhanced T cell killing when co-cultured with pre-activated T cells (Supplementary Figure S1D). Thus, TRIM32 in tumor cells promotes M2-like TAM polarization and T cell dysfunction.

### TRIM32 interacts with PDE9A

**Fig. 3:**
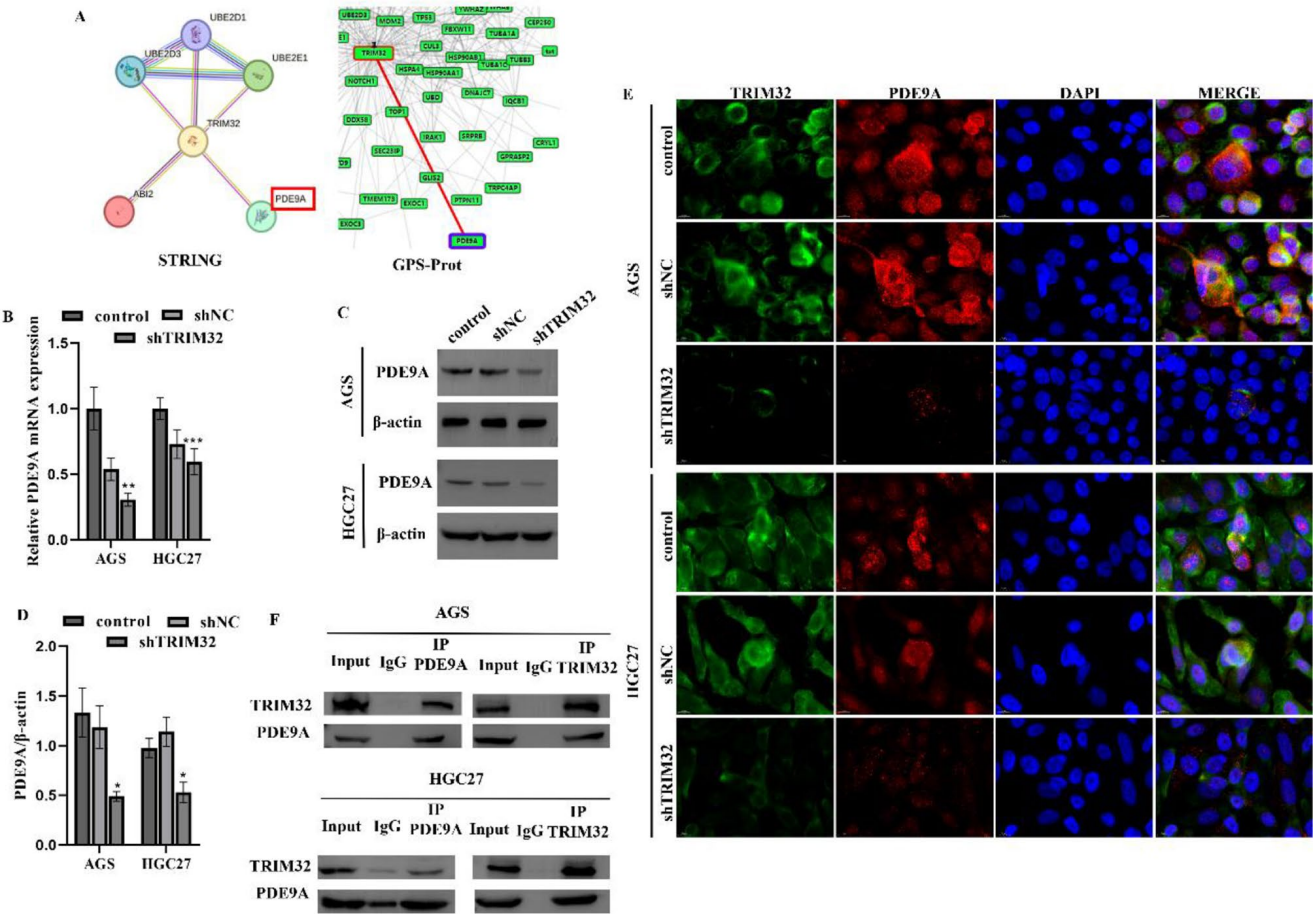
TRIM32 interacts with PDE9A. (**A**) STRING and GPS-Prot database indicated TRIM32 may interact with PDE9A. (**B-D**) TRIM32 knockdown in AGS and HGC27 cells decreased the expression level of PDE9A by quantitative PCR (**B**), and western blotting. Fluorescence colocalization (**E**, scale = 10 μm) and Co-IP (**F**) analysis of the interactions between TRIM32 and PDE9A in AGS and HGC27 cells. Data with error bars are shown as mean ± SD. *P < 0.05, ***P < 0.001 vs. control

To investigate the relationship between TRIM32 and TAMs infiltration, we performed interaction prediction and transcriptome analysis. PDE9A garnered our attention due to its interaction with TRIM32 (Fig. 3A), and transcriptome sequencing revealed significantly decreased expression levels in the shTRIM32 group (Supplementary file 2). Research has demonstrated that PDE9A is implicated in the pathogenesis of various tumors and cancers [24, 25] and can selectively hydrolyze cyclic guanosine monophosphate (cGMP) [26]. Modulating the cGMP-protein kinase G (PKG) signaling pathway can affect the aggressive development of GC and encourage the conversion of macrophages into pro-inflammatory M1 phenotypes [27]. Consequently, we hypothesize that TRIM32 may regulate macrophage polarization via PDE9A.

Validation through qRT-PCR and WB confirmed the reduced expression of PDE9A in HGC27 or AGS/shTRIM32 cells (Fig. 3B-D). We examined TRIM32’s impact on endogenous PDE9A activity and found that TRIM32 knockdown decreased PDE9A activity (Supplementary Figure S2). Immunofluorescence staining was employed to detect the interaction of TRIM32 with PDE9A. As shown in Fig. 3E, shTRIM32 inhibited PDE9A accumulation in AGS and HGC27 cells, as shown by the co-localization of TRIM32 with PDE9A.To further validate the immunofluorescence results, we conducted a Co-IP assay. As shown in Fig. 3F, the interaction of endogenous TRIM32 with endogenous PDE9A.

### TRIM32/PDE9A axis promoted recruitment and M2 polarization of macro-phages

**Fig. 4:**
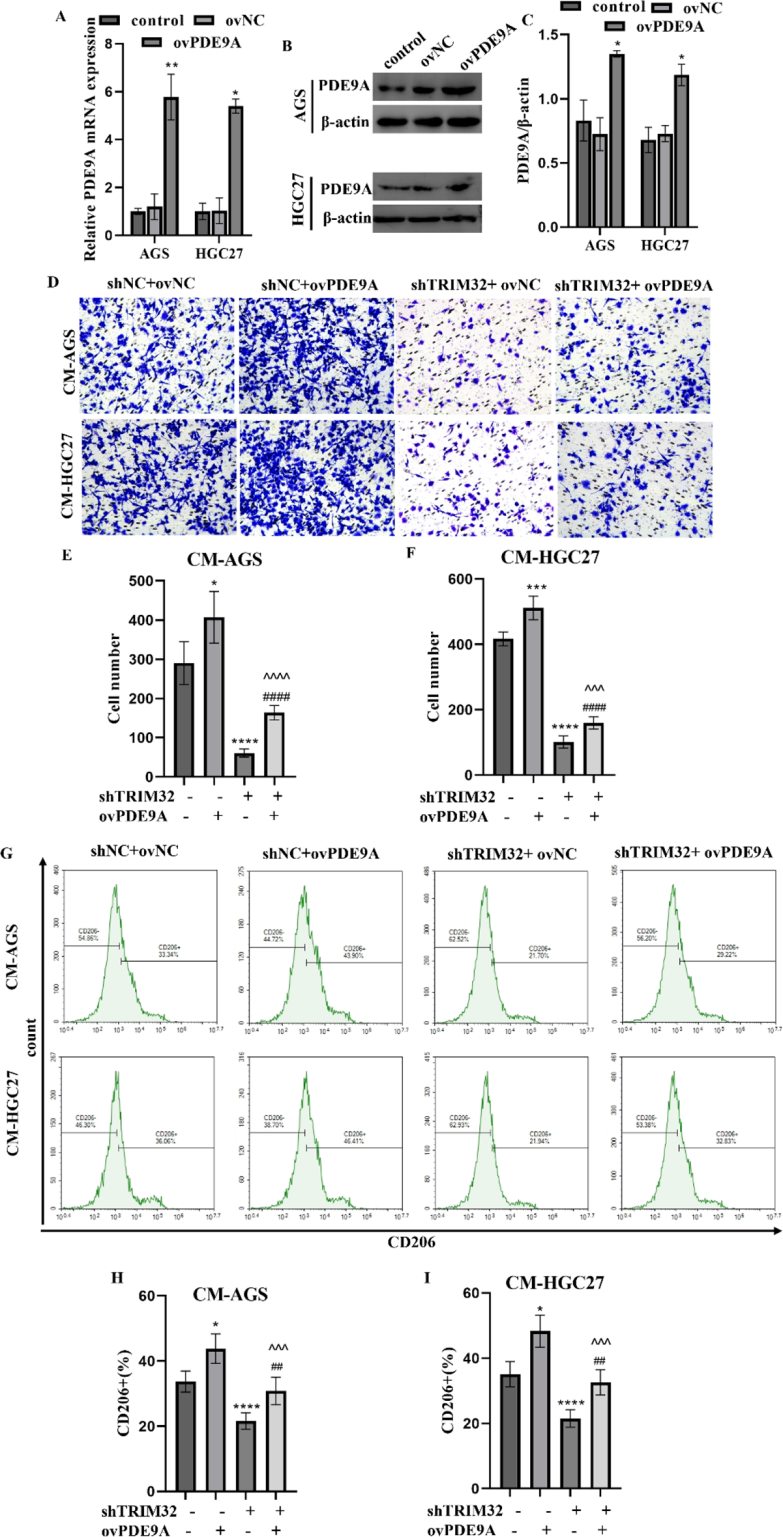
TRIM32/PDE9A axis enhances macrophage recruitment and M2 polarization in vitro. (**A-C**) PDE9A expression significantly increased in AGS and HGC27 cells following transfection with a PDE9A-overexpressing lentivirus, as shown by qRT-PCR (**A**) and western blotting (**B-C**). Tumor cells were first co-transfected with lentivirus carrying TRIM32 shRNA and PDE9A for 48 h, and CM was collected for macrophage culture and TAM polarization. (**D-F**) Overexpression PDE9A reversed TRIM32 knockdown and increased macrophage migration. The migration ability of TAMs was evaluated by the transwell migration assay. (**D**) Images of migrated cells and (**E-F**) average results from each group. (**G-I**) Overexpression PDE9A reversed TRIM32 knockdown and increased M2-like macrophage polarization. The polarization ability of macrophage cells was assessed by (**G**) flow cytometry results and (**H-I**) average results from 3 independent experiments. *P < 0.05, **P < 0.01, ***P < 0.001, ****P < 0.0001 vs. control. ^^^*P* < 0.001, ^^^^*P* < 0.0001 vs. shTRIM32 + ovNC. ^##^*P* < 0.01, ^####^*P* < 0.0001 vs. shNC + ovPDE9A

To advance our understanding of the regulatory mechanism by which GC TRIM32 influences TAMs, we established HGC27 and AGS GC cell lines with stable PDE9A overexpression (Fig. 4A-C). Transwell (Fig. 4D-F) and flow cytometry assays (Fig. 4G-I) showed that PDE9A overexpression significantly promoted cell migration capacities of THP-1 and M2 polarization of macrophages. In order to explore the functions of TRIM32/PDE9A in the recruitment and M2 polarization of macrophages, a rescue assay was conducted. Transwell (Fig. 4D-F) revealed that PDE9A overexpression efficiently restored the migration capacities of THP-1 cells in CM-AGS/HGC27. Similarly, flow cytometry (Fig. 4G-I) showed that the inhibitory effects on M2 macrophage polarization induced by shTRIM32 were reversed by the overexpression of PDE9A.

### The PI3K/AKT signaling pathway activation by TRIM32/PDE9A is responsible for the M2 polarization of macrophages

We examined how TRIM32 promotes an immunosuppressive macrophage phenotype. RNA-seq analysis of AGS/shNC and AGS/shTRIM32 cells showed 4041 genes with altered expression after TRIM32 knockdown (false discovery rate < 0.05, Log_2_FC > 1.5) (Supplementary Figure S3A). The cluster map analysis indicated that the knockout of TRIM32 led to a substantial reduction in the expression levels of numerous genes (Supplementary Figure S3B). KEGG pathway analysis indicated that the PI3K/AKT pathway was the most enriched among the downregulated genes (Supplementary Figure S4A). GSEA and expression correlation analysis further linked TRIM32 to PI3K/AKT signaling in AGS (Supplementary Figure S4B). We hypothesized that TRIM32 activates the PI3K/AKT pathway by regulating PDE9A expression. WB confirmed that TRIM32 overexpression increased p-p85 and p-AKT levels, while PDE9A knockdown decreased this change induced by TRIM32 overexpression (Supplementary Figure S5).

**Fig. 5:**
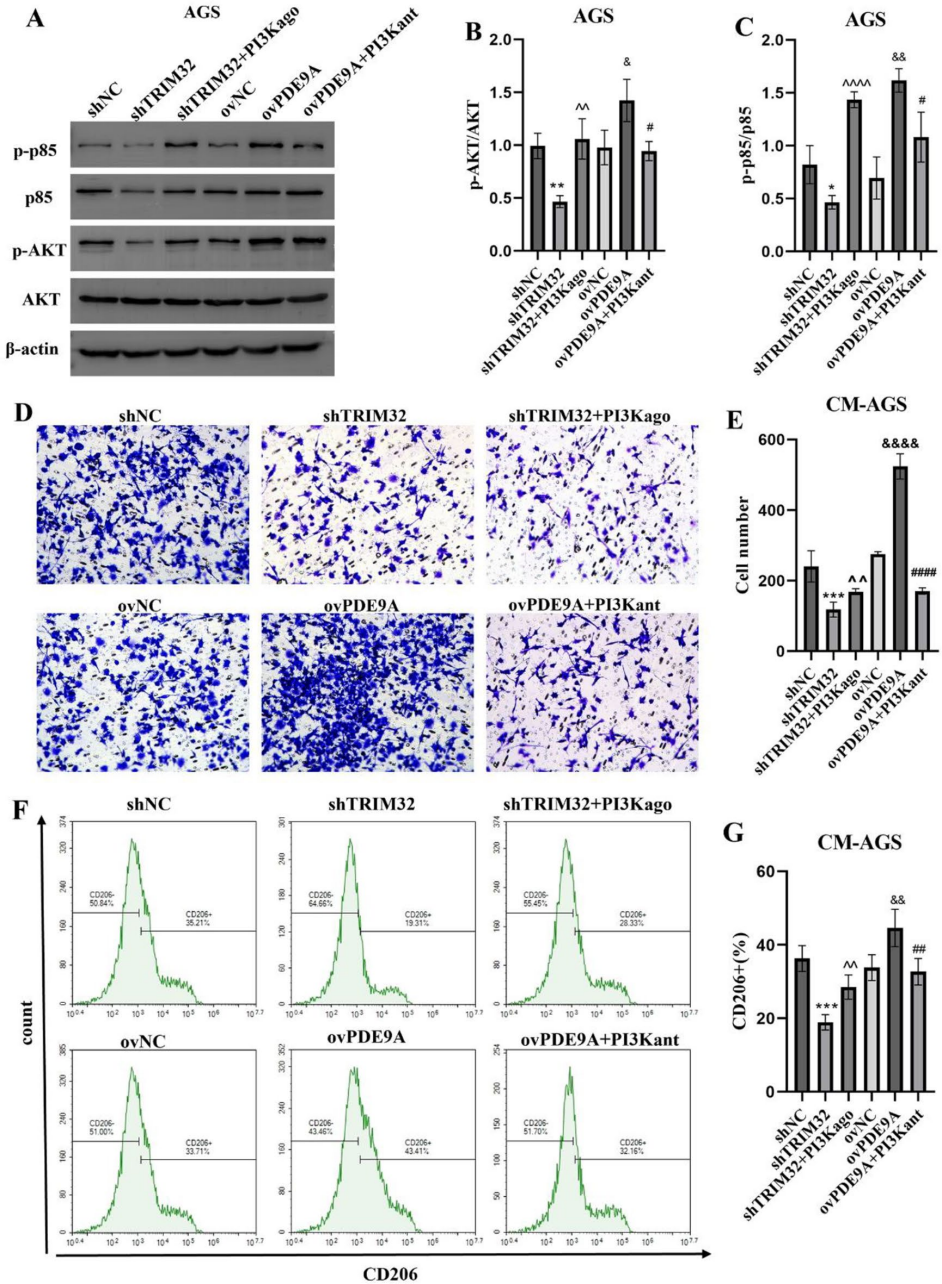
The PI3K/AKT signaling activation by TRIM32/PDE9A is responsible for the M2 polarization of macrophages in vitro. (**A-C**) TRIM32 and PDE9A modulate the PI3K/AKT signaling pathway in AGS cells. (**A**) Representative western blotting bands and (**B-C**) quantitative analysis of the bands. (**D-E**) PI3Kant effectively inhibited macrophage migration stimulated by the overexpression of PDE9A, whereas PI3Kago restored macrophage migration that was suppressed due to TRIM32 knockdown. Tumor cells were first transfected with lentivirus carrying TRIM32 shRNA or PDE9A for 48 h. CM was then collected for macrophage culture and TAM polarization. TAM migration was evaluated using a transwell migration assay. (**D**) Images of migrated cells were taken, and (**E**) average results from each group were shown. (**F-G**) PI3Kant effectively inhibited M2-like macrophage polarization stimulated by the overexpression of PDE9A, whereas PI3Kago rescued M2-like macrophage polarization that was suppressed due to TRIM32 knockdown. The polarization ability of macrophage cells was assessed by (**F**) flow cytometry results and (**G**) average results from three independent experiments. Data with error bars are shown as mean ± SD. ***P < 0.001 vs. control. ^^*P* < 0.01 vs. shTRIM32. ^&&^*P* < 0.01, ^&&&&^*P* < 0.0001 vs. ovNC. ^##^*P* < 0.01, ^####^*P* < 0.0001 vs. ovPDE9A

Next, we examined if the PI3K/AKT pathway was responsible for TRIM32-induced immunosuppressive reprogramming of macrophages. TRIM32 knockdown inhibited pathway-related genes (p-p85 and p-AKT), while PDE9A overexpression increased their expression. Using PI3K activator IGF-1 and inhibitor IPI-549, we found IGF-1 restored PI3K/AKT activity suppressed by TRIM32 deficiency, and IPI-549 countered the activation by PDE9A (Fig. 4A-C, Supplementary Figure S6A-C). Conversely, IPI-549 treatment reversed the phosphorylation increase caused by PDE9A overexpression, re-inhibiting the PI3K/AKT pathway. Moreover, the results of rescue assays revealed that IGF-1 or IPI-549 treatment completely reversed the effects of shTRIM32 and ovPDE9A on macrophage migration and M2 macrophage polarization (Fig. 4 D-G, Supplementary Figure S6D-G). Altogether, TRIM32 activated PI3K/AKT pathway to accelerate macrophage migration and M2 macrophage polarization partially via a PDE9A-mediated manner (Fig. 5).

### TRIM32 knockdown reduces TAMs in GC in vivo

**Fig. 6:**
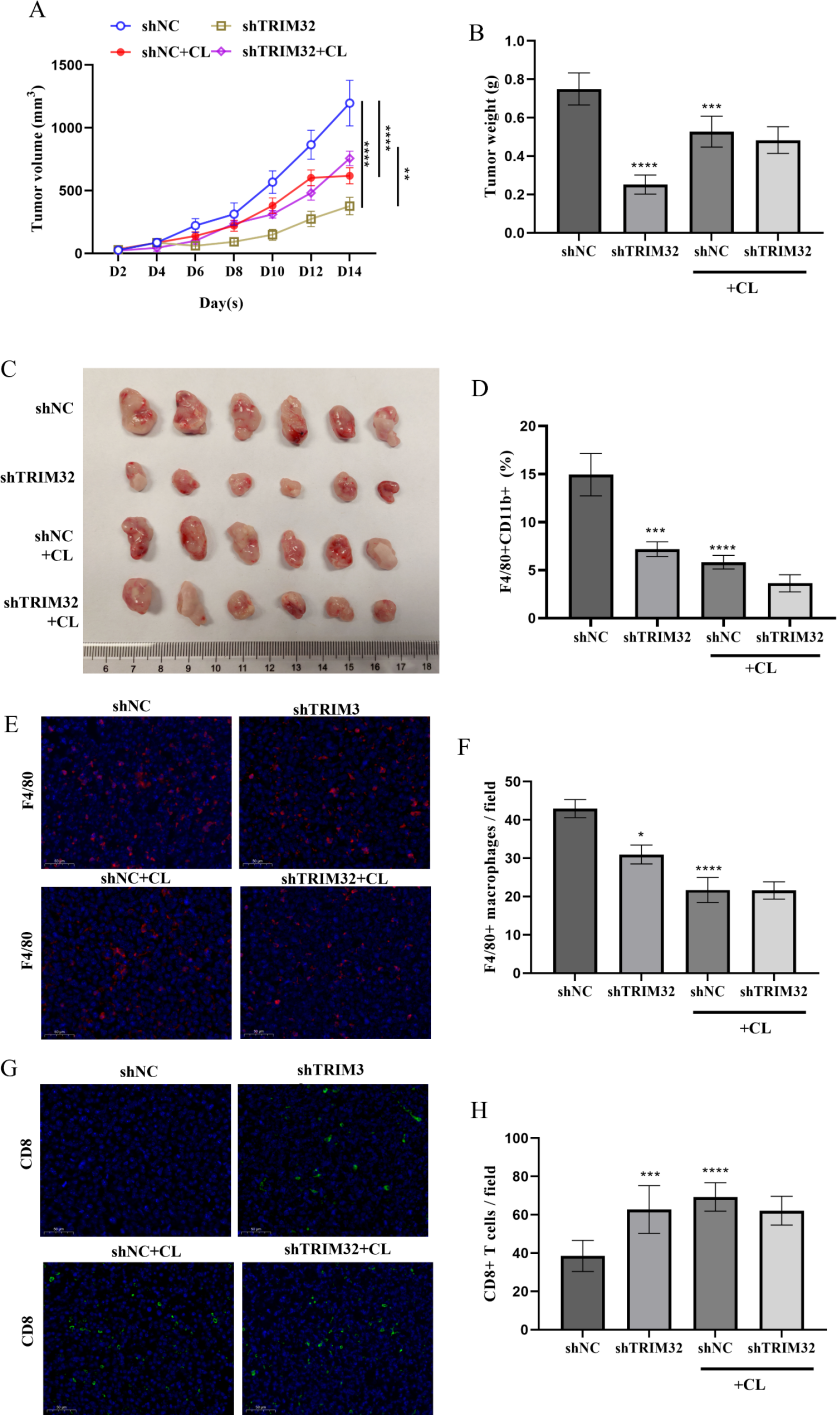
Impaired macrophage recruitment and tumor growth in vivo upon shTRIM32 of mice with GC. (**A-C**) TRIM32 knockdown or CL treatment markedly inhibited tumor growth in BALB/C nude mice, while CL treatment eliminates the difference caused by TRIM323 knockdown. (**A**) Tumor growth rate in each group. (**B**) tumor weight of BALB/C mice treated with CL, shTRIM32 or shNC (*n* = 6 per group). (**C**) Tumor images obtained from each mouse on day 14. (**D-F**) TRIM32 knockdown or CL treatment markedly inhibited F4/80 + and CD206 macrophages, while CL treatment eliminates the difference caused by TRIM323 knockdown. (**D**) Flow cytometry analysis of the proportions of infiltrated F4/80 + CD11b + macrophages in tumor. (E**-F**) TRIM32 knockdown or CL treatment markedly inhibited F4/80 + macrophages, while CL treatment eliminates the difference caused by TRIM323 knockdown. (**E**) Representative images of F4/80 + macrophages infiltration. (**F**) Average results from 3 independent. (G**-H**) TRIM32 knockdown or CL treatment markedly increased CD8 + T cells, while CL treatment eliminates the difference caused by TRIM323 knockdown. (**G**) Representative images of CD8 + T cells (scale = 10 μm). (**H**) Average results from three independent. *P < 0.05, ***P < 0.001, ****P < 0.0001 vs. shNC

To further investigate the impact of TRIM32 knockdown on TAMs, we used CL to deplete macrophages in GC model mice. Tumor volume and weight measurements were taken to assess whether the reduced tumor growth observed with TRIM32 knockdown was due to diminished macrophage recruitment. The results showed that shTRIM32 led to a significant decrease in tumor volume and weight. Interestingly, tumors in the shNC group were notably larger than those in the shTRIM32 group, but this difference was nullified by CL treatment (Fig. 6A-C). We conducted flow cytometry to analyze macrophage proportions in tumors. The results indicated a significant reduction of TAMs (F4/80 + CD11b + cells) in the shTRIM32 groups, and CL treatment effectively decreased TAM levels (Fig. 6D, Supplementary Figure S7). CL treatment depleted TAMs successfully. Furthermore, immunofluorescence results showed a further reduction in TAMs (F4/80 + cells) in the shTRIM32 groups (Fig. 6E-F), and higher CD8 + T cells in the shTRIM32 groups compared to the shNC group (Fig. 6G-H). However, this disparity was no longer observed under the conditions of CL treatment, which suggested the increase in CD8 + T cells may be an adaptive response to macrophage depletion.

### Inhibition of TRIM32 reduce the drug resistance of anti-PD-1 therapy

The modulation of tumor immunity is influenced by PD-1 expression within TAMs [28]. Firstly, we found that levels of CD274 (encoding PD-L1, a ligand of PD-1) were lower in AGS/HGC27 cells with TRIM32 knockdowns (Supplementary Figure S8A and B). Then, the level of CD274 in AGS/HGC27/shTRIM32 cells co-cultured with THP-1 was lower than that with other cells (Supplementary Figure S8C and D). Furthermore, the level of CD279 (encoding PD-1) in THP-1 cells co-cultured with AGS/HGC27/shTRIM32 was lower than that THP-1 co-cultured with AGS/HGC27/control, or AGS/HGC27/shNC cells (Supplementary Figure S8E and F). The above results prompted us to ask whether TRIM32 is sufficient to cause anti-PD-1 resistance.

**Fig. 7:**
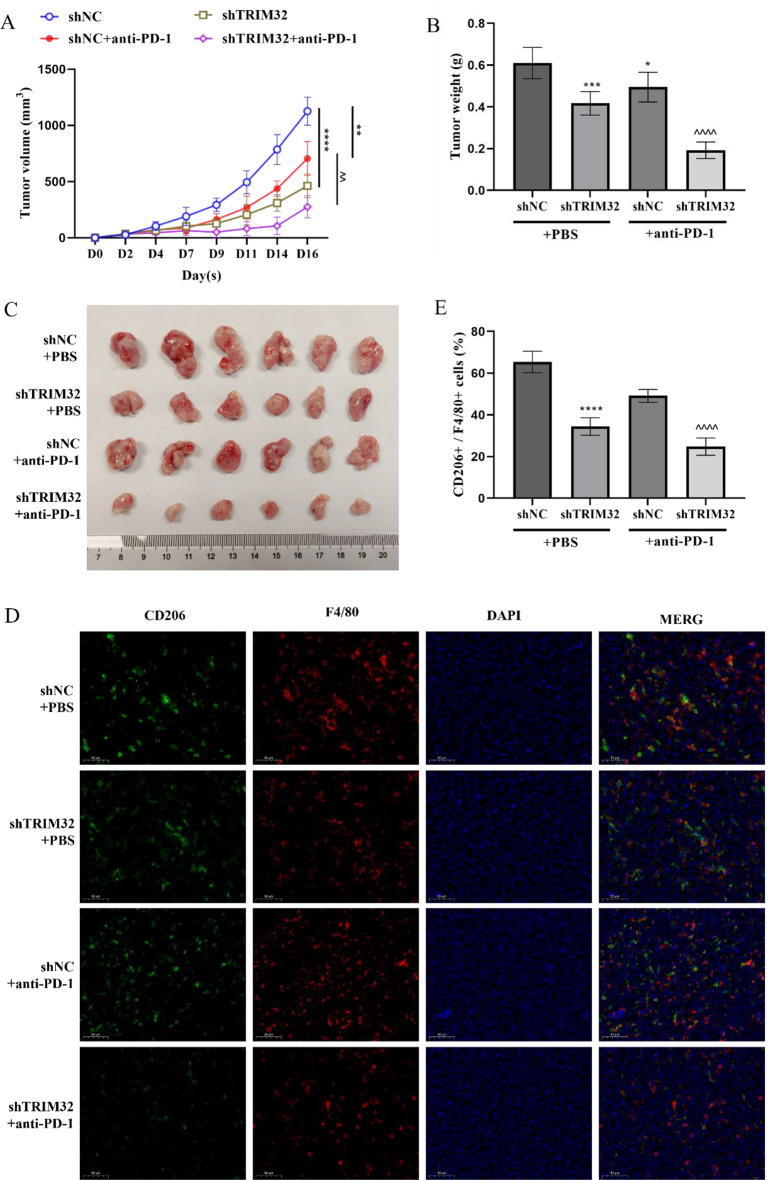
The knockdown of TRIM32 enhances the efficacy of anti-PD1 therapy in GC. (**A**) TRIM32 knockdown or PD-1 neutralization slowed tumor growth in BALB/C nude mice, but the combination of both treatments had the greatest effect. Tumor growth rates for each treatment are shown. (**B**) Tumors in the TRIM32 knockdown and PD-1 inhibition groups weighed less than controls, with the lowest weights in the combined treatment group. A histogram shows the mean ± SD of tumor weights at the study’s end (*n* = 6 mice/group). (**C**) Tumor images obtained from each mouse on day 16. (**D-E**) TRIM32 knockdown and/or PD-1 antibody treatment significantly reduced F4/80 + and CD206 + macrophages, with the fewest in the combined treatment group. This was also seen in the CD206+/F4/80 + ratio. (**D**) Immunofluorescence images. (**E**) Quantification of macrophages via immunofluorescence. *P < 0.05, ***P < 0.001, ****P < 0.0001 vs. shNC + PBS. ^^*P* < 0.01, ^^^^*P* < 0.0001 vs. shTRIM32 + anti-PD-1

In order to explore whether TRIM32 can reduce anti-PD-1 resistance, we constructed a mouse model of GC with blocking of TRIM32, and anti-PD-1 antibody was administered intraperitoneally. We observed a trend of increased PD-1 and PD-L1 expression in immune cells of shTRIM32 mice, though it wasn’t statistically significant (Supplementary Figure S8G-H). However, shTRIM32 significantly inhibited tumor growth, and the combination of shTRIM32 with anti-PD-1 therapy led to a more pronounced reduction in tumor volume and weight compared to shTRIM32 alone (Fig. 7A-C). PD-1/PD-L1 resistance is associated with M2-like TAMs [29]. For the TME analysis, we observed that shTRIM32 decreased tumoral infiltration of M2 macrophages. However, the reduction in M2 macrophages was more significantly in the shTRIM32 combined with anti-PD-1 treatment group (Fig. 7D-E).

## Discussion

This study demonstrated that TRIM32 is linked to a worse prognosis in GC. By interacting with PDE9A, TRIM32 initiates the downstream PI3K/AKT signaling pathway, which facilitates the recruitment of TAM and encourages M2-like polarization. This process ultimately creates an immunosuppressive microenvironment within GC, reducing the effectiveness of anti-PD-1 treatment. These results offer valuable insights into the mechanisms of macrophage infiltration in GC.

Evidence shows that M2-like macrophages play a crucial role in the advancement of several types of cancer, including melanoma, lung, and GC [30, 31]. TAMs hinder cytotoxic lymphocyte function, facilitating tumor immune escape [32–34]. A positive relationship was observed between the expression of TRIM32 and the presence of TAMs in GC tissues, as well as in online data sources. Knockdown of TRIM32 inhibits the recruitment of macrophages and the polarization towards an M2-like phenotype. In a GC mouse model, tumors in the shNC group exhibited a notably quicker growth rate compared to those in the shTRIM32 group, but this difference was eliminated by CL treatment. CL treatment can also remove the disparity between the decline of macrophages and the increase of T cells due to TRIM32 knockout. These results underscore the essential function of tumor TRIM32 expression as a pivotal regulator of immunosuppression within the TME, thereby offering a robust justification for the therapeutic targeting of this pathway.

This study shows that TRIM32 interacts with PDE9A, a gene that regulates cGMP homeostasis. PDE9A decreases PKG activity through the hydrolysis of cGMP, whereas the stimulation of cGMP/PKG signaling can suppress Wnt/β-catenin transcription, inhibit the proliferation of cancer cells, and enhance tumor immunity [35]. Huang et al. showed that ADH1A promotes macrophage transformation to the M1 phenotype via the cGMP/PKG pathway in GC [27]. In this study, the overexpression of PDE9A rescued the inhibitory effects of shTRIM32 on the migration of macrophages and the polarization to M2 macrophages. TAM polarization is a key factor in cancer’s immune escape and growth, contributing to tumor progression and treatment failure [36–38]. Thus, blocking the TRIM32-PDE9A-cGMP-PKG axis may represent a potential approach for tumor management. Evidence indicates that inhibiting cGMP PDE is linked to the cancer growth suppression seen with sulindac and other nonsteroidal anti-inflammatory drugs (NSAIDs) [39]. Currently, while there are clinical drugs targeting PDE9A, including BAY-7081 [40] and PF-04447943 [41], their application in cancer treatment remains unexplored. Our findings offer a theoretical foundation for the potential application of these drugs in cancer; however, clinical validation is necessary.

The PI3K/AKT signaling pathway is vital for modulating the functions of both immune and tumor cells [42]. In the context of macrophages, PI3K/AKT/mTOR signaling is believed to influence macrophage transcription [43]. Furthermore, activation of the PI3K/AKT pathway has been shown to enhance macrophage-mediated immune evasion within the TME [44]. Our research indicates that TRIM32 interacts with PDE9A to stimulate downstream PI3K/AKT pathway, an essential component in the generation of M2 macrophages. Inhibiting PI3K has been found to decrease the presence of M2 macrophages. Our research confirms the significance of PI3K/AKT pathway in the induction of M2 macrophages by TRIM32, enhancing our comprehension of its impact on tumor immunity.

Anti-PD-1 immunotherapy has been used for GC patients, but many show poor response to PD-1 antibodies alone [45]. To enhance effectiveness, combining anti-PD-1 with chemotherapy, targeted therapy, or radiation is suggested. This study assessed the synergistic impact of dual blockade of TRIM32 and PD-1 in GC models, revealing that TRIM32 knockout enhances the effectiveness of anti-PD-1 therapy. Immunofluorescence analysis showed a significant reduction in M2 macrophages in tumor tissues treated with shTRIM32 + anti-PD-1. This is similar to what has been found that M2 macrophages in the TME are key players in mediating the resistance against anti-PD-1 therapy in GC [45]. These findings propose that TRIM32 may serve as a promising target for combination therapy alongside anti-PD-1 treatments. Although, no direct TRIM32 inhibitor is currently available, however, it provides a theoretical basis for future targeted combined anti-PD-1 therapy.

Nude mice are commonly used to study cancer’s immune microenvironment [46], but their lack of a functional thymus impairs T-cell responses, limiting immune escape research. At the same time, depletion of TAMs using CL may non-specifically affect other macrophage populations. Consequently, it is imperative to construct a diverse array of GC mouse models in future research to validate these findings. Although interspecies differences exist, numerous fundamental molecular pathways implicated in GC are conserved between mice and humans. Elucidating the key molecules and their interactions within mouse models could reveal potential therapeutic targets for the treatment of human GC [47, 48]. But more clinical samples are necessary to better clarify the diagnostic or therapeutic roles of TRIM32. The research also has some other limitations. The 2D in vitro models employed in this study may not adequately recapitulate the complexity of the TME. We have not determined whether TRIMM32 interacts directly or indirectly with PDE9A. While previous research has indicated that TAM can impact the cytotoxic activity of CD8 + T cells and natural killer (NK) cells on tumor cells, as well as contribute to cancer-related inflammation [4], these findings were not confirmed in our study.

The study revealed a beneficial relationship between the expression of TRIM32 and the TAM in patients with GC. It was demonstrated that the TRIM32/PDE9A-PI3K/AKT pathway enhances tumor suppression by inducing M2-like polarization of macrophages within the TME. Additionally, downregulation of TRIM32 was shown to decrease resistance to anti-PD-1 therapy. These findings suggest that targeting TRIM32 could improve the efficacy of anti-PD-1 treatment in GC, highlighting its potential as an immunotherapy target.

## Electronic supplementary material

Below is the link to the electronic supplementary material.

Supplementary Material 1

Supplementary Material 2

## Data Availability

The data that support the findings of this study are available from the corresponding author upon reasonable request.

## Abbreviations

GC: Gastric cancer
TME: Tumor microenvironment
TRIM32: Tripartite motif 32
TAMs: Tumor-associated macrophages
PDE9A: Phosphodiesterase 9 A
PI3K/Akt: Phosphatidylinositol-3-kinase/protein kinase B
MFC: Mouse forestomach carcinoma
FBS: Fetal bovine serum
CM: Conditioned medium
NC: Negative control
qRT-PCR: Quantitative reverse transcription–polymerase chain reaction
WB: Western blotting
KM-plot: Kaplan⁃Meier Plotter
Co-IP: Co-immunoprecipitation
