# Supplementary material for: TRIM32 promotes tumor immune evasion and impedes Anti–PD-1 treatment by inducing immunosuppressive macrophages in gastric cancer
Source: J Transl Med. 2025 Oct 29;23:1187. doi: 10.1186/s12967-025-06330-8 (PMC12574041; doi:10.1186/s12967-025-06330-8)
Supplement: Supplementary file 2 — Supplementary Material 2 [file 12967_2025_6330_MOESM2_ESM.docx]

**Supplementary material**

### Generation of human T cells and T cell cytolytic assay

Peripheral blood mononuclear cells (PBMCs) from healthy volunteers were isolated through Ficoll-Paque density centrifugation of freshly donated whole blood. Naïve CD3+ T cells were obtained using EasySep T cell enrichment kits (StemCell Technologies; Cat No. 19751). Flow cytometry confirmed that the purity of the naïve T cells exceeded 97%. The T cell fractions underwent stimulation with 50 U/ml IL2 and CD3/CD28 Dynabeads (Thermo Fisher Scientific, Waltham, MA, USA), utilizing a bead-to-cell ratio of 1:8 over a period of 48 hours.

T cells were incubated together with target cells (AGS) at various effector to target cell ratios (1:1, 5:1, and 10:1) in triplicate within 96-well plates for a duration of 24 hours. The cytolytic capacity of T cells was evaluated utilizing a lactate dehydrogenase (LDH) release assay kit (Biyotime, China). LDH concentrations and the absorbance values in the culture supernatants were used to quantify the cytolytic activity of T cells. The specific cytotoxicity percentage was determined using the formula: Cytotoxicity = (Experimental LDH release - Spontaneous LDH release) / (Maximal LDH release - Spontaneous LDH release) × 100%. Here, the experimental LDH release refers to the LDH that was liberated during the co-culture of effector and target cells, while spontaneous release denotes the LDH emitted from tumor cells in the absence of effector cells. Maximal LDH release corresponds to the LDH levels obtained after the addition of Triton X-100 (indicating 100% LDH release) to the cells.

### Determination of PDE9A

### Immunoprecipitates derived from transfected cells were washed three times with PBS and once with PDE assay buffer (10 mM Tris–HCl, pH 7.4) before being utilized for the PDE assay. The measurement of PDE activity was conducted using the cyclic nucleotide PDE assay kit (Enzo Life Sciences) in accordance with the provided instructions.

### Immunohistochemistry analysis

An immunohistochemical evaluation was performed to specifically analyze the expression of PD-1 and PD-L1 within the tumor tissue. The procedure utilized 4µm-thick sections of formalin-fixed paraffin-embedded (FFPE) tumor tissue. The sections underwent deparaffinization using xylene and were rehydrated through a sequential ethanol gradient into water. To retrieve antigens, slides were heated in a sodium citrate buffer (10 mM, pH 6) for 10 minutes at 100°C for both PD-1 and PD-L1. Endogenous peroxidase activity was inhibited by treating the sections with a 3% hydrogen peroxide solution in either methanol or distilled water for 10 minutes at room temperature. Following this, the sections were exposed overnight at 4 °C in a humidified chamber to monoclonal rabbit anti-PD-L1 (1:200, clone E1L3N, Cell Signaling Technology) and monoclonal mouse anti-PD-1 (1:20, clone MRQ-22, Cell Marque), diluted in an antibody diluent. After rinsing with PBS, the slides were incubated at 37 °C for 30 minutes with universal immuno-peroxidase polymer anti-Rabbit or anti-Mouse antibodies (#H2008, #2004, NICHIREI BIOSCIENCES Inc. Tokyo, Japan). Visualization of antibody binding was achieved using bright 3,3′-diaminobenzidine (DAB) (ImmunoLogic) for 7 minutes at room temperature. Finally, the slides were counterstained with hematoxylin, dehydrated, and covered with a coverslip.

**Supplementary Table 1** **Antibody information**

| **Antibody** | **Application** | **Species Reactivity** | **Dilution** | **Supplier** | **Catalog number** |
| --- | --- | --- | --- | --- | --- |
| Anti-CD204 | Flow cytometry | Human | 1:50 | Abcam | ab271070 |
| Anti-CD206 | Flow cytometry | Human | 1:50 | Abcam | ab270647 |
| Anti-CD206 | Flow cytometry | Mouse | 1:50 | Thermo Fisher | 17-2061-82 |
| Anti-F4/80 | Flow cytometry | Mouse | 1:50 | Thermo Fisher | 11-4801-82 |
| Anti-CD11b | Flow cytometry | Mouse | 1:50 | Thermo Fisher | 12-0112-82 |
| Anti-PDE9A | WB | Human | 1:500 | Abcam | ab97556 |
| Anti-TRIM32 | WB | Human | 1:500 | Abcam | ab96612 |
| Akt | WB | Human, mouse | 1:1000 | Cell Signaling Technology | #9272 |
| phosphorylated (p)-Akt | WB | Human, mouse | 1:2000 | Cell Signaling Technology | #4060 |
| p-p85 | WB | mouse | 1:1000 | Cell Signaling Technology | #17366 |
| β-actin | WB | Human, mouse | 1:1000 | Cell Signaling Technology | #4967 |
| HRP Anti-Rabbit IgG antibody | WB | Rabbit | 1:2000 | Abcam | ab288151 |
| Anti-CD8 antibody | IF | Mouse | 1:1000 | Thermo Fisher | MA1-10301 |
| Anti-F4/80 antibody | IF | Human, mouse | 1:100 | ThermoFisher | MA1-91124 |
| Anti-CD206 | IF | Mouse, Rat, Human | 1:1000 | Abcam | ab64693 |
| Anti-CD68 | IF | Human | 1:50 | Abcam | ab283316 |
| Anti-PDE9A | WB, IF | Human | 1:100 | Abcam | ab168432 |
| Alexa Fluor 555-conjugated anti-mouse IgG | IF | mouse | 1:500 | Thermo Fisher | A32794 |
| Alexa Fluor 555-conjugated anti-rat IgG | IF | Rat | 1:500 | Abcam | ab150158 |
| Alexa Fluor 488-conjugated anti-rabbit antibody | IF | rabbit | 1:500 | Thermo Fisher | A-11008 |
| Anti-PDE9A | IF | Human | 1:100 | Abcam | ab168432 |
| anti-TRIM32 | Co-IP | human, mouse, rat | 1:500 | Proteintech | 10326-1-AP |
| anti-PDE9A | Co-IP | human, mouse, rat | 1:1000 | Santa Cruz | sc-376271 |

**Supplementary Table 2** Association between TRIM32 expression and clinicopathologic characteristics in gastric cancer patients.

| **Clinical parameter** | TRIM32 expression | | P value |
| --- | --- | --- | --- |
|  | High (n=59) | Low (n=59) |  |
| **Age (years)** |  |  | 0.709 |
| ≥60 | 24 | 26 |  |
| <60 | 35 | 33 |  |
| **Gender** |  |  | 0.205 |
| Male | 47 | 41 |  |
| Female | 12 | 18 |  |
| **Tumor size** |  |  | 0.001 |
| ≥5cm | 49 | 33 |  |
| <5cm | 10 | 26 |  |
| **TNM stage** |  |  | 0.018 |
| I-II | 13 | 25 |  |
| III-IV | 46 | 34 |  |
| **Differentiation** |  |  | 0.004 |
| Well | 0 | 8 |  |
| Moderate | 9 | 14 |  |
| Poor | 50 | 37 |  |
| **Depth of invasion** |  |  | 0.364 |
| Mucous layer | 1 | 1 |  |
| Muscular layer | 8 | 14 |  |
| Serosal layer | 50 | 44 |  |


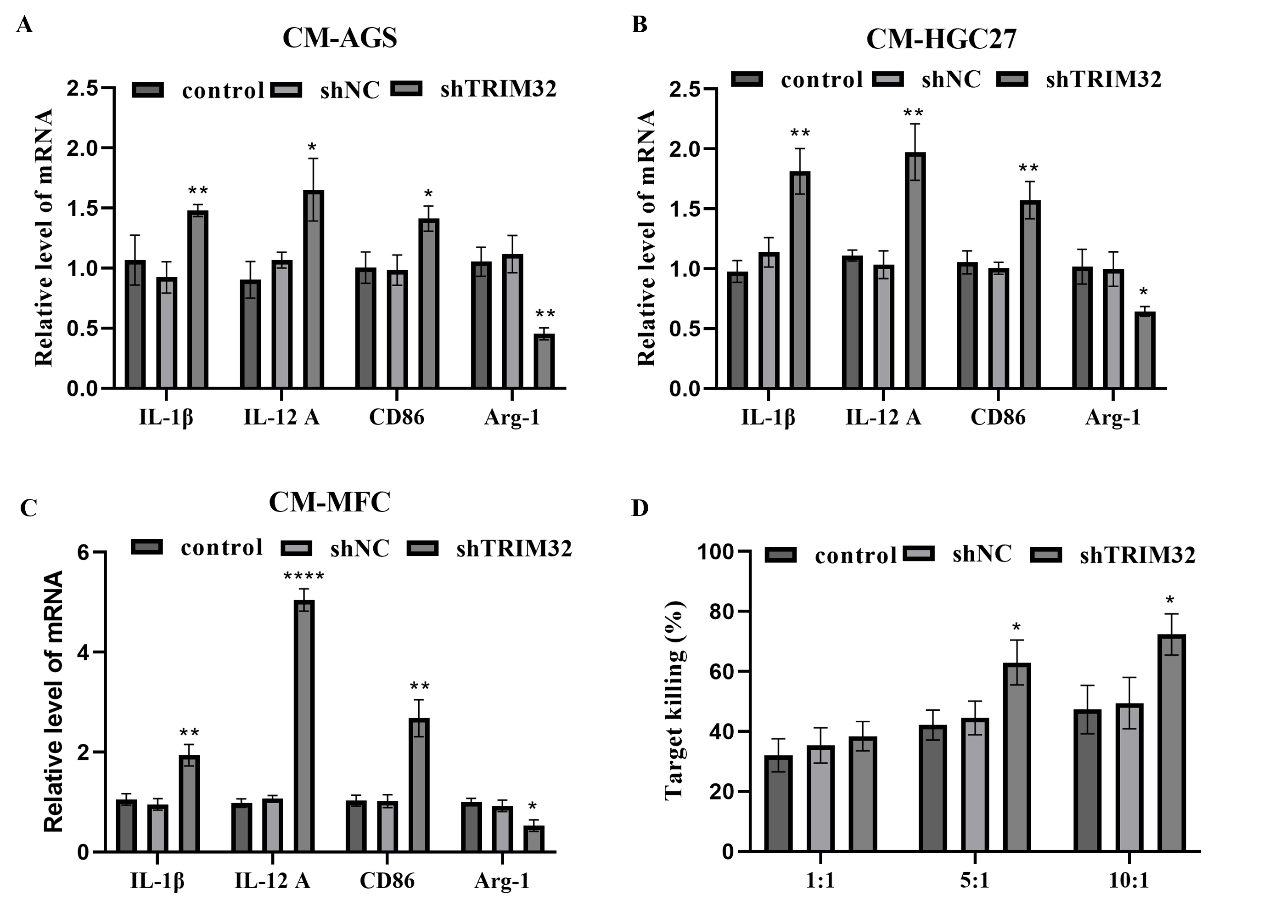


Figure S1. TRIM32 knockdown regulates macrophage polarization and enhances T cell killing ability. (A-B) qRT-PCR evaluations of Arg-1, IL-1β, IL-12a, and CD86 were performed on THP-1 cells exposed to the CM from AGS/HGC27 cells for 24 h. (C) qRT-PCR evaluations of Arg-1, IL-1β, IL-12a, and CD86 in RAW264.7 treated with the CM from MCF cells for 24 h. (D) T cells co-cultured with TRIM32-knockdown AGS cells demonstrated enhanced cytolytic activity. T cells were co-cultured with TRIM32-downregulated or control AGS cells at different E: T ratios for 4 days, and the cytolysis assay was used to assess the killing ability of T cells. E:T ratio: Effector: target cell ratio; *P < 0.05, ***P < 0.001, ****P < 0.0001 vs. control.


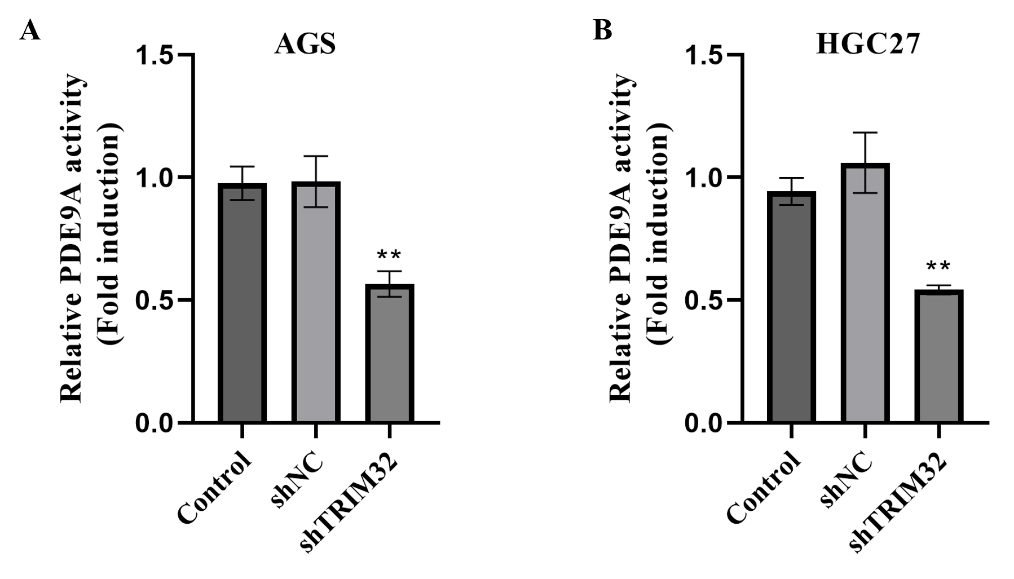


Figure S2. TRIM32 knockdown inhibited PDE9A enzyme activity. (A-B) AGS and HGC27 cell lysates with TRIM32 knockdown were immunoprecipitated using an anti-PDE9A antibody and assessed for PDE activity. **P < 0.01 vs. control.


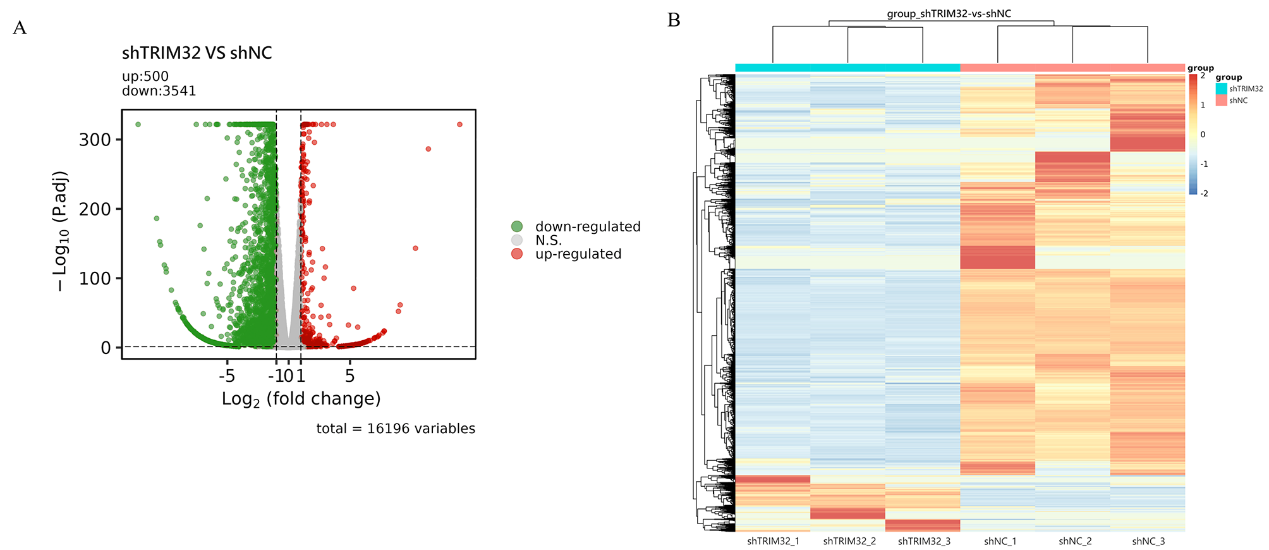


Figure S3. Differential expression analysis after TRIM32 knockdown. (A) Volcano plot of differentially expressed genes post-TRIM32 depletion. (B) Hierarchical clustering heatmap.


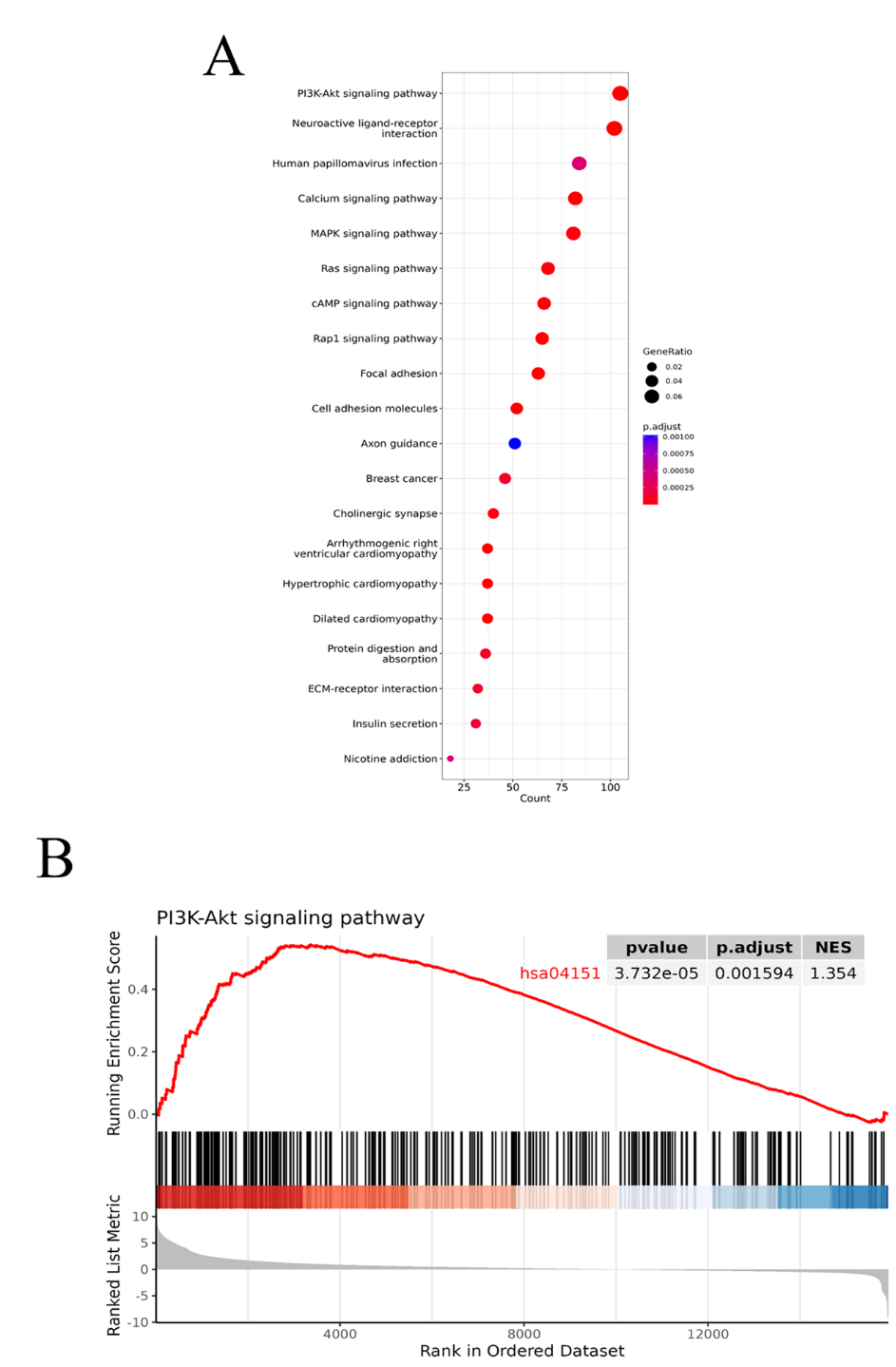


Figure S4. Enrichment analysis of downregulated genes due to TRIM32 knockdown. (A) KEGG analysis identified the top 20 enriched pathways in AGS cells with shTRIM32. (B) GSEA and expression correlation analysis revealed a strong association between TRIM32 and PI3K/AKT signaling in AGS.


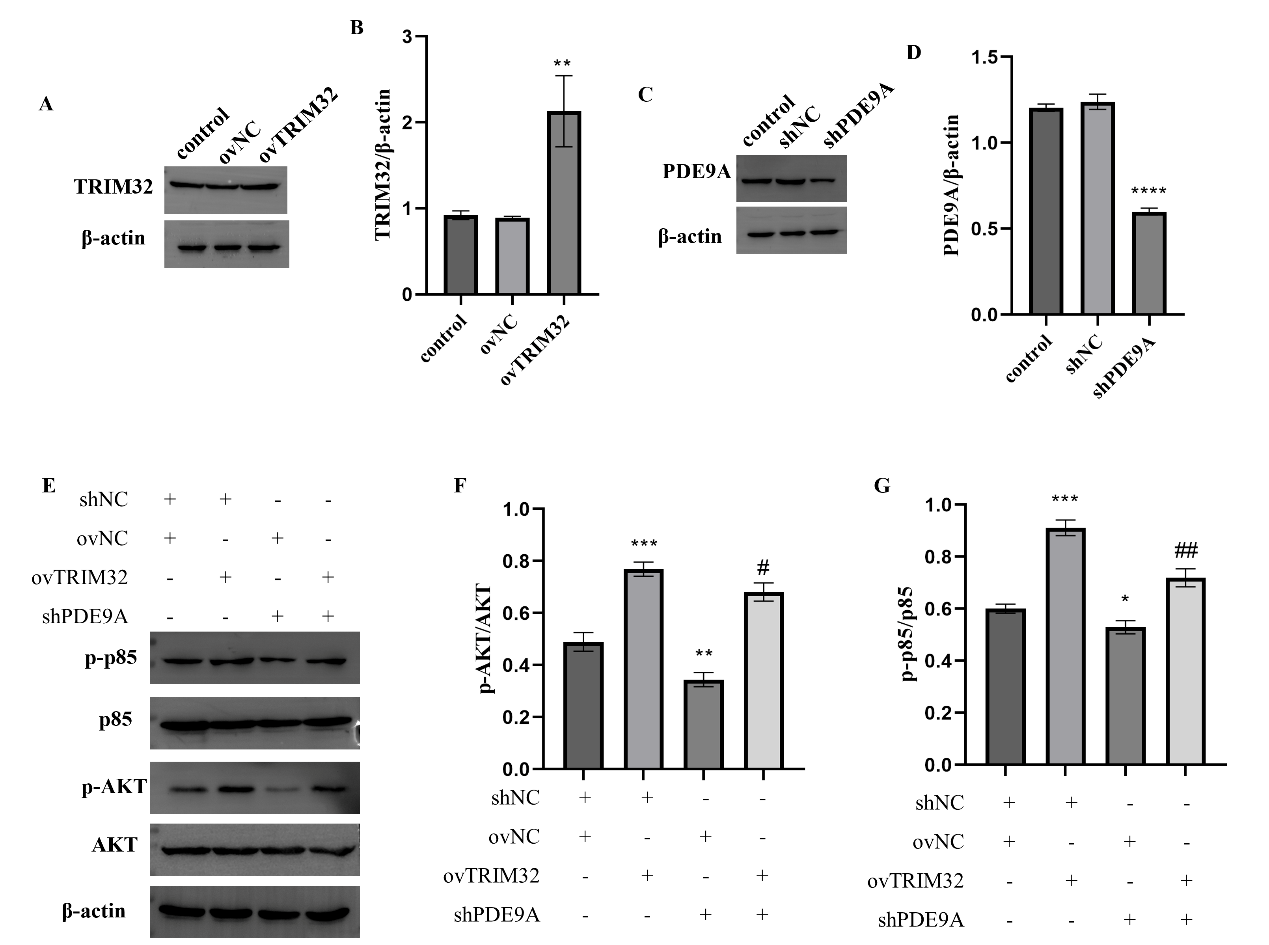
Figure S5. The PI3K–AKT signaling activation by TRIM32/PDE9A. (A-B) The expression of TRIM32 was significantly upregulated in AGS cells following transfection with a lentivirus containing specific TRIM32 sequences, as confirmed by Western blot analysis. (C-D) Conversely, the expression of PDE9A was markedly downregulated in AGS cells upon transfection with a lentivirus carrying specific shRNA sequences targeting PDE9A, as demonstrated by Western blotting. (E-G) The modulation of the PI3K/AKT signaling pathway by TRIM32/PDE9A in AGS cells was further elucidated. (E) Representative Western blot bands and (F-G) quantitative analyses of these bands are presented. *P < 0.05, ***P < 0.001, ****P < 0.0001 vs. shNC+ovNC or control. #P < 0.05, ##P < 0.01 vs. ovTRIM32+shNC.


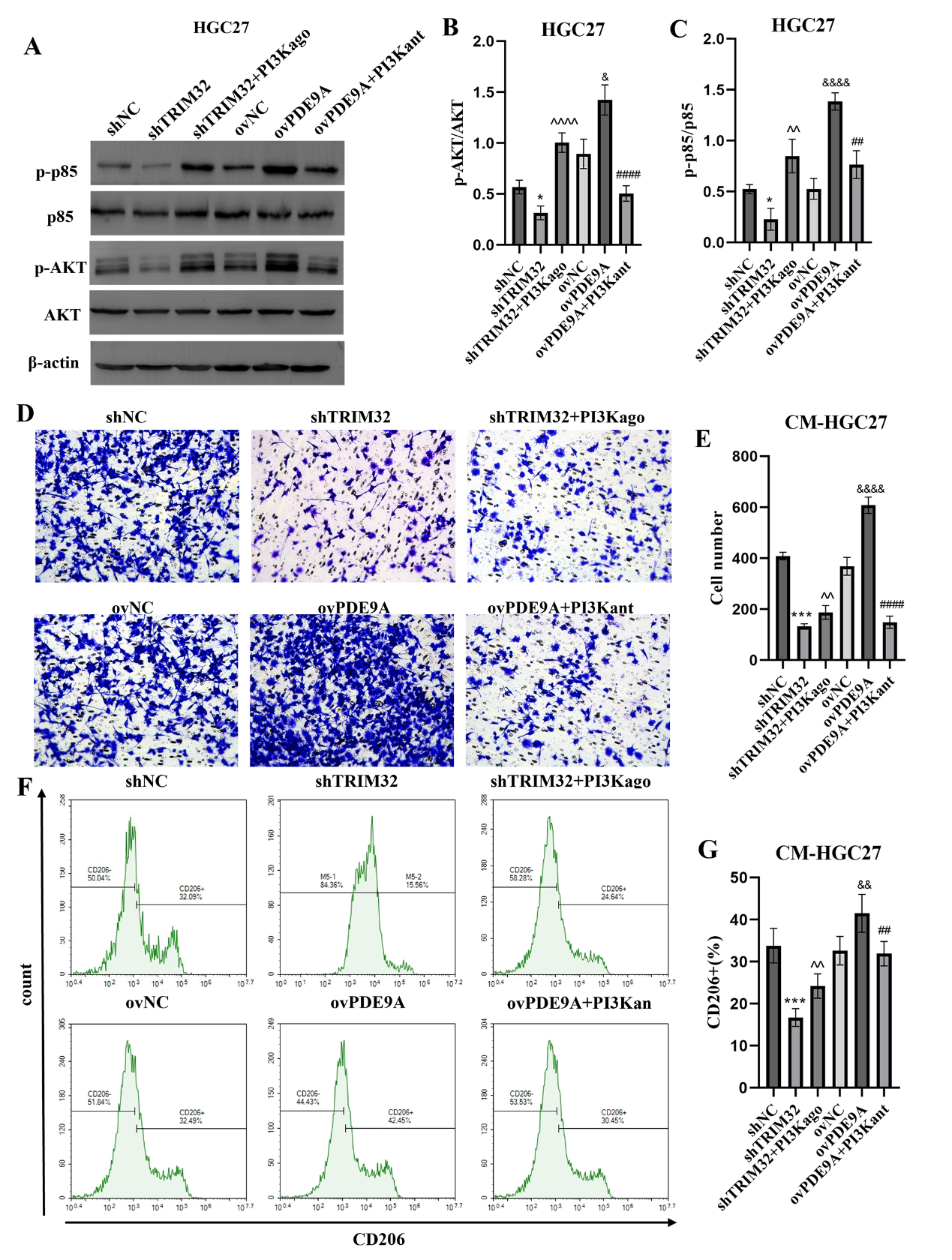
Figure S6. The PI3K–AKT signaling activation by TRIM32/PDE9A is responsible for the M2 polarization of macrophages in vitro. (A-C) Western blot analysis of p-P85, P85, p-Akt, Akt, and β-actin in HGC27 cells. (D-E) PI3Kant effectively inhibited macrophage migration stimulated by the overexpression of PDE9A, whereas PI3Kago restored macrophage migration that was sup-pressed due to TRIM32 knockdown. Tumor cells were initially transfected with lentivirus that contained TRIM32 shRNA or PDE9A for a duration of 48 h. Following this, CM was gathered for the purpose of culturing macrophages and polarizing TAMs. The migratory capacity of TAMs was assessed using the Transwell migration assay. (D) Photographs of the migrated cells and (E-F) average outcomes from three separate experiments are presented. (F-G) PI3Kant effectively inhibited M2-like macrophage polarization stimulated by the overexpression of PDE9A, whereas PI3Kago rescued M2-like macrophage polarization that was suppressed due to TRIM32 knockdown. The capability of macrophage cells to polarize was evaluated using (F) flow cytometry data and (G) mean outcomes from three separate experiments. Data with error bars are shown as mean ± SD. ***P < 0.001 vs. control. ^^P < 0.01 vs. shTRIM32. &&P < 0.01, &&&&P < 0.0001 vs. ovNC. ##P < 0.01, ####P < 0.0001 vs. ovPDE9A.


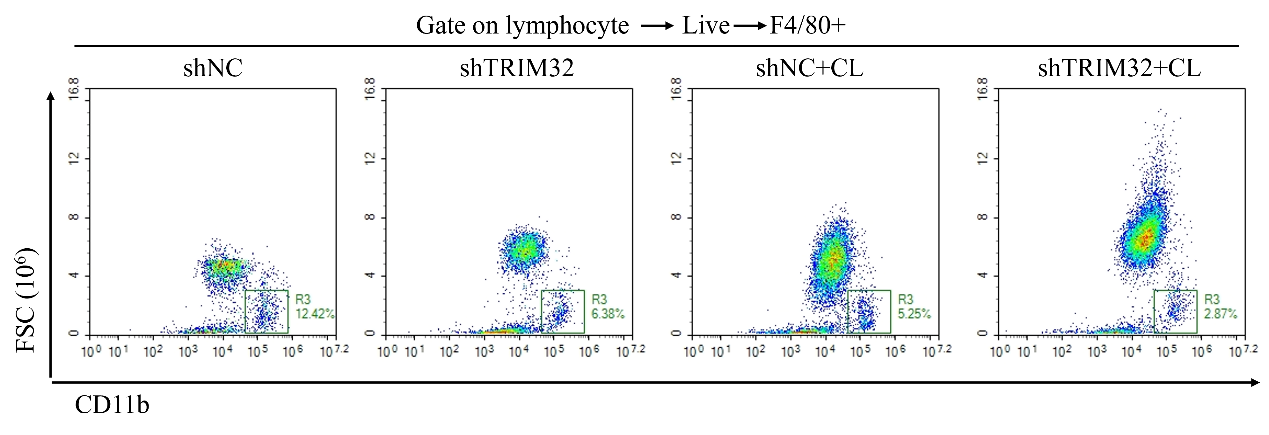


Figure S7. Gating strategy used for intratumoral F4/80+CD11b+ macrophages. FACS gating strategy of F4/80+CD11b+ macrophages is shown for Fig. 6D.


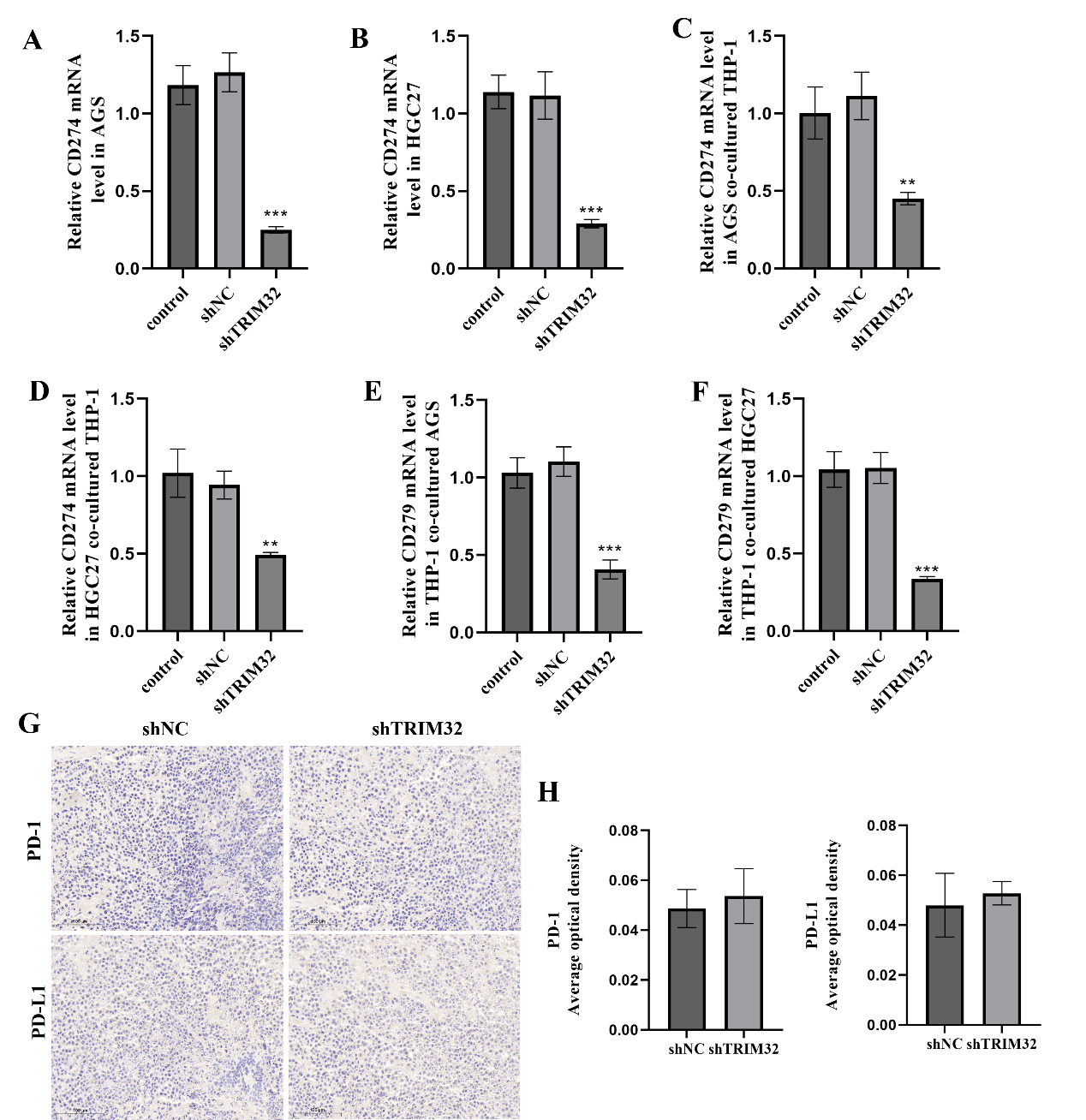


Figure S8. TRIM32 knockdown suppresses the PD-1/PD-L1 pathway in vitro. (A-B) AGS/HGC27 cells expressing control, shNC, and shTRIM32. qRT-PCR was performed for CD274. (C-F) Monocytes THP-1 were co-cultured for 48 h with AGS/HGC27 cells expressing control, shNC, or shTRIM32. CD274 expression in AGS/HGC27 cells (C-D) and CD279 mRNA levels in THP-1 cells (E-F) were assessed using qPCR. *P < 0.05, ***P < 0.001, ****P < 0.0001 vs. control. (G-H) Immunohistochemical staining and quantification of PD-1 and PD-L1 in mouse tumors. *P < 0.05, ***P < 0.001, ****P < 0.0001 vs. shNC.
